# Mechanistic Insights on Salicylic Acid-Induced Enhancement of Photosystem II Function in Basil Plants under Non-Stress or Mild Drought Stress

**DOI:** 10.3390/ijms25115728

**Published:** 2024-05-24

**Authors:** Ilektra Sperdouli, Emmanuel Panteris, Julietta Moustaka, Tuğba Aydın, Gülriz Bayçu, Michael Moustakas

**Affiliations:** 1 Institute of Plant Breeding and Genetic Resources, Hellenic Agricultural Organisation–Demeter (ELGO-Dimitra), 57001 Thermi, Greece; esperdouli@elgo.gr; 2 Department of Botany, Aristotle University of Thessaloniki, 54124 Thessaloniki, Greece; epanter@bio.auth.gr; 3 Department of Food Science, Aarhus University, 8200 Aarhus, Denmark; julietta_moustaka@food.au.dk; 4 Department of Biology, Faculty of Science, Istanbul University, 34134 Istanbul, Turkey; taydin1@ogr.iu.edu.tr (T.A.); gulrizb@istanbul.edu.tr (G.B.)

**Keywords:** chlorophyll fluorescence imaging, non-photochemical quenching, electron transport rate, plastoquinone pool, photoprotective heat dissipation, chloroplast ultrastructure

## Abstract

Photosystem II (PSII) functions were investigated in basil (*Ocimum basilicum* L.) plants sprayed with 1 mM salicylic acid (SA) under non-stress (NS) or mild drought-stress (MiDS) conditions. Under MiDS, SA-sprayed leaves retained significantly higher (+36%) chlorophyll content compared to NS, SA-sprayed leaves. PSII efficiency in SA-sprayed leaves under NS conditions, evaluated at both low light (LL, 200 μmol photons m^−2^ s^−1^) and high light (HL, 900 μmol photons m^−2^ s^−1^), increased significantly with a parallel significant decrease in the excitation pressure at PSII (1-*qL*) and the excess excitation energy (EXC). This enhancement of PSII efficiency under NS conditions was induced by the mechanism of non-photochemical quenching (NPQ) that reduced singlet oxygen (^1^O_2_) production, as indicated by the reduced quantum yield of non-regulated energy loss in PSII (Φ*_NO_*). Under MiDS, the thylakoid structure of water-sprayed leaves appeared slightly dilated, and the efficiency of PSII declined, compared to NS conditions. In contrast, the thylakoid structure of SA-sprayed leaves did not change under MiDS, while PSII functionality was retained, similar to NS plants at HL. This was due to the photoprotective heat dissipation by NPQ, which was sufficient to retain the same percentage of open PSII reaction centers (q*p*), as in NS conditions and HL. We suggest that the redox status of the plastoquinone pool (q*p*) under MiDS and HL initiated the acclimation response to MiDS in SA-sprayed leaves, which retained the same electron transport rate (ETR) with control plants. Foliar spray of SA could be considered as a method to improve PSII efficiency in basil plants under NS conditions, at both LL and HL, while under MiDS and HL conditions, basil plants could retain PSII efficiency similar to control plants.

## 1. Introduction

The significant shifts in climate patterns, leading to more frequent and intense drought-stress (DS) episodes and the high light (HL) intensity in the Mediterranean region, have a detrimental impact on crop yield [1,2,3,4]. Water scarcity stands as the primary environmental stressor affecting various physiological processes in plants, thereby influencing global agricultural output [1,5,6,7]. With climate change, the frequency, intensity, and duration of DS episodes are projected to escalate, affecting plant performance across all growth stages from germination to maturity [8,9]. Drought stress disrupts critical plant functions such as cell division, elongation, and differentiation, disturbs osmotic balance, and impairs photosynthesis, ultimately diminishing productivity [10,11,12].

Under DS, there is an imbalance between the absorbed light energy and its utilization in photosynthesis [13,14,15]. When the light energy absorbed by antenna overdoes the photosynthetic capability, it over-reduces the electron transport chain, therefore leading to the creation of harmful reactive oxygen species (ROS) [16,17]. This excess absorbed light energy must be dissipated as heat by the non-photochemical quenching (NPQ) mechanism to prevent the formation of ROS [18,19,20,21,22,23,24]. ROS production, including the superoxide anion radical (O_2_^•^^−^), hydrogen peroxide (H_2_O_2_), and singlet oxygen (^1^O_2_), rises due to the disruption of ROS homeostasis under drought stress [25,26,27,28]. This imbalance triggers oxidative stress, resulting in membrane damage, protein degradation, and enzyme inactivation, further compromising cellular integrity [3,29,30,31,32].

Stomatal closure under drought stress reduces CO_2_ availability and electron usage, consequently having to divert electrons from the electron transport chain to oxygen, generating O_2_^•^^−^ [29]. Additionally, energy transfer from the triplet state chlorophylls (^3^Chl*) to oxygen (O_2_) produces ^1^O_2_, which can further produce other ROS (O_2_^•^^−^, H_2_O_2_) [18,28,33,34,35], exacerbating membrane damage [36,37,38,39]. The combined effects of drought stress and intense sunlight radiation pose a severe threat to crop production, accelerating ROS production [36,37,40,41,42]. However, under DS, modulation of chlorophyll synthesis and a reduction in light-harvesting complexes can mitigate excess light absorption, thus lowering ROS production [43]. Plants with reduced chlorophyll content and smaller antenna sizes absorb less light energy, minimizing ROS creation [44]. Therefore, decreasing leaf chlorophyll content is proposed as a strategy to alleviate photooxidative stress, particularly in Mediterranean climates with HL intensity [44,45,46,47,48,49,50].

Salicylic acid (SA), a phenolic compound and essential plant hormone, plays a crucial role in stress defense and growth regulation [51,52]. Increased SA production, along with decreased auxin biosynthesis, coordinates plant defense responses, mitigating the adverse effects of drought and salinity stress by improving physiological parameters, membrane integrity, and photosynthetic efficiency [51,52,53,54,55]. The impact of SA on plants varies depending on plant species and the experimental conditions [50,56]. Foliar application of 1 mM SA in tomato plants has been shown to mitigate phototoxicity by reducing chlorophyll content and protecting photosystem II (PSII), thereby improving photosynthetic function [50]. Enhancing photosynthesis and light energy utilization can increase plant productivity [57].

The effects of externally applied SA on plant physiological functions under non-stress (NS) conditions remain absonant, with some studies suggesting a positive impact, while others indicating a negative impact on various physiological processes [58]. In soybean and corn, for instance, foliar application of SA led to increased photosynthetic rates, likely due to enhanced enzyme activity associated with CO_2_ uptake, rather than changes in stomatal aperture [58,59]. Conversely, when 0.5 mM SA was added to maize plants, it resulted in decreased photosynthesis accompanied by reductions in stomatal conductance (gs) and transpiration rate [60]. However, this same concentration of SA has been shown to offer protection against low-temperature-induced damage in young maize plants [60] or in mitigating the harmful effects of paraquat in barley [61]. SA has been recognized to have a constructive role on plant acclimation to many abiotic stresses, such as chilling, drought, heavy metal toxicity, heat, and salinity [50,62,63,64,65,66,67,68,69,70,71]. The dissimilar effects of SA on diverse plant species might be due to diversification of its biosynthesis and signaling pathways in different plants [72]. SA in plants is biosynthesized from chorismate through two pathways: (i) isochorismate synthase and (ii) phenylalanine ammonia lyase [73,74,75].

*Ocimum basilicum* L., is a member of the Lamiaceae family, commonly known as basil. Basil plants are widely cultivated, being one of the most extensively consumed culinary, aromatic, and medicinal plant in many countries [76,77,78]. Over the past few decades, there has been a rise to the incorporation of herbal crops like basil into daily diets, attributed to their potential in preventing certain cancers, cardiovascular issues, and other chronic diseases, due to their abundant levels of essential oils and phenolic compounds [76].

Exogenously applied SA in oregano seedlings has been reported to enhance PSII functionality under NS conditions only under HL and not under low light (LL) [79]. However, under mild drought stress (MiDS), it improved PSII photochemistry at both LL and HL conditions [79]. Since it has been suggested that the mode of action of SA varies with plant species [50,56,79], we evaluated the responses of basil plants to exogenously applied SA under the same experimental conditions. Thus, the aim of this study was to distinguish any differences in the molecular mechanisms of light energy partitioning at PSII of basil plants, with or without foliar application of SA and grown under NS or MiDS conditions. The hypothesis tested in our work was that the application of SA would enhance PSII functionality in basil plants under NS conditions at both LL and HL and ameliorate the negative impact of MiDS on PSII functionality, also under both LL and HL.

## 2. Results

### 2.1. Chlorophyll Content and Leaf Water Content under Non-Stress or Mild Drought Stress with or without Salicylic Acid

**Figure 1:**
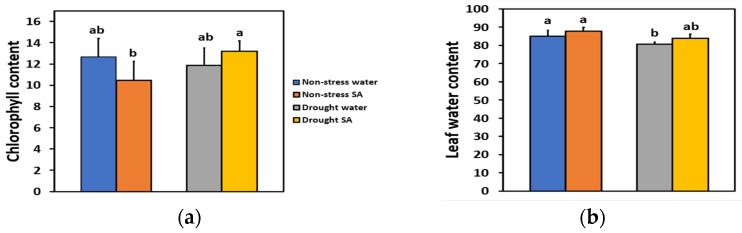
Chlorophyll content (relative units) (**a**) and leaf water content (%) (**b**) of basil plants 48 h after the spray with water or 1 mM SA under non-stressed (NS) conditions or 96 h after withholding the irrigation on the same plants (MiDS), so the soil volumetric water content (SWC) reached 60% of the well-watered plants (NS). Error bars are standard deviations (SD). Different lowercase letters express significant difference.

We estimated the chlorophyll content and the leaf water content of basil plants 48 h after spraying with distilled water or with 1 mM SA. We also estimated the chlorophyll content on the same plants that were subjected to MiDS by withholding the irrigation for 96 h, so the soil volumetric water content (SWC) reached 60% of the SWC of non-stressed (well-watered control) plants. The chlorophyll content of basil leaves under NS or MiDS conditions did not differ between water-sprayed or SA-sprayed leaves (Figure 1a). However, under MiDS, SA-sprayed leaves retained significantly higher (+36%) chlorophyll content compared to NS SA-sprayed leaves (Figure 1a).

Leaf water content under NS or MiDS conditions did not differ between water-sprayed or SA-sprayed basil plants (Figure 1b). Mild drought-stress (MiDS) treatment led to a significantly lower leaf water content of water-sprayed basil leaves compared to both non-stressed, water-sprayed (−5%), or SA-sprayed (−8%) plants (Figure 1b). In contrast, under MiDS, the leaf water content of SA-sprayed leaves did not differ compared to non-stressed, water-sprayed, or SA-sprayed plants (Figure 1b).

### 2.2. Light Energy Partitioning at Photosystem II under Non-Stress or Mild Drought Stress with or without Salicylic Acid

**Figure 2:**
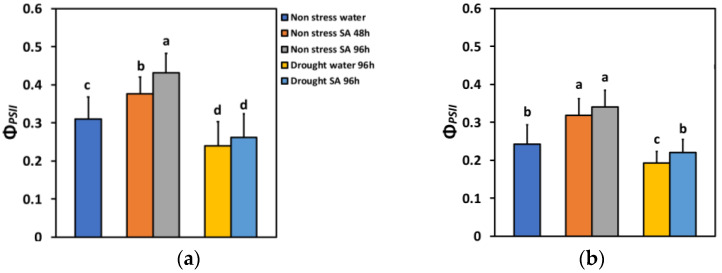
The effective quantum yield of PSII photochemistry (Φ*_PSII_*) at 200 μmol photons m^−2^ s^−1^ (LL) (**a**) or 900 μmol photons m^−2^ s^−1^ (HL) (**b**) of basil plants, under non-stressed control conditions (water sprayed), and 48 h and 96 h after the spray with 1 mM SA, or 96 h after withholding the irrigation on the water-sprayed or SA-sprayed plants. Error bars are standard deviations (SD). Different lowercase letters express significant difference.

The absorbed light energy by the light harvesting complexes (LHC) at PSII is either allocated to photochemistry (Φ*_PSII_*), or it is dissipated as heat (Φ*_NPQ_*), or it is lost by a nonregulatory way (Φ*_NO_*) [80]. The total of all these three fluxes is equal to one [80]. In non-stressed (NS) basil plants that were sprayed with SA, this partitioning of energy under low light (LL), compared to water-sprayed ones (controls), changed significantly. Forty-eight hours after SA spraying, the effective quantum yield of PSII photochemistry (Φ*_PSII_*) increased by 21%, while after 96 h, it increased by 39% (Figure 2a). Under high light (HL), Φ*_PSII_* increased even more after SA spraying (31% after 48 h and 40% after 96 h) (Figure 2b).

Φ*_PSII_* under MiDS conditions and LL decreased significantly compared to NS conditions in both water-sprayed (−23%) or SA-sprayed (−16%) plants but without any significant difference between the two treatments (Figure 2a). In contrast, under HL, despite a significant decrease in Φ*_PSII_* under MiDS in water-sprayed (−21%) plants, the basil plants that were sprayed with 1 mM SA retained the same Φ*_PSII_* with non-stressed (control) plants (Figure 2b). SA-sprayed basil plants retained significantly higher Φ*_PSII_* (+14%) compared to water sprayed, under MiDS treatment (Figure 2b).

**Figure 3:**
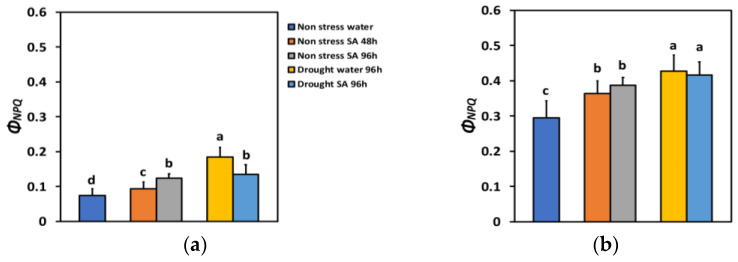
The quantum yield of regulated non-photochemical energy loss in PSII (Φ*_NPQ_*) at 200 μmol photons m^−2^ s^−1^ (LL) (**a**) or 900 μmol photons m^−2^ s^−1^ (HL) (**b**) of basil plants, under non-stressed control conditions (water sprayed), and 48 h and 96 h after the spray with 1 mM SA, or 96 h after withholding the irrigation on the water-sprayed or SA-sprayed plants. Error bars are standard deviations (SD). Different lowercase letters express significant difference.

The quantum yield of regulated non-photochemical energy loss in PSII (Φ*_NPQ_*) 48 h after SA spraying increased by 26% and after 96 h, by 67%, which is significantly higher than the 48 h treatment period (Figure 3a). Under high light (HL), Φ*_NPQ_* increased by 23% 48 h after SA spraying and after 96 h, by 31% (Figure 3b). However, there was not any significant difference between the two time periods (Figure 3b).

Φ*_NPQ_* under MiDS conditions and LL increased significantly in both water-sprayed (+148%) or SA-sprayed (+81%) plants compared to NS water-sprayed plants (Figure 3a). Thus, in SA-sprayed leaves, the increase in Φ*_NPQ_* under MiDS was lower by 27% compared to water-sprayed leaves (Figure 3a). Under MiDS and HL, Φ*_NPQ_* increased less (by 45% in water-sprayed leaves and by 41% in SA-sprayed leaves, compared to NS water-sprayed plants) (Figure 3b). However, there was not any significant difference in Φ*_NPQ_* between the two different treatments (Figure 3b).

**Figure 4:**
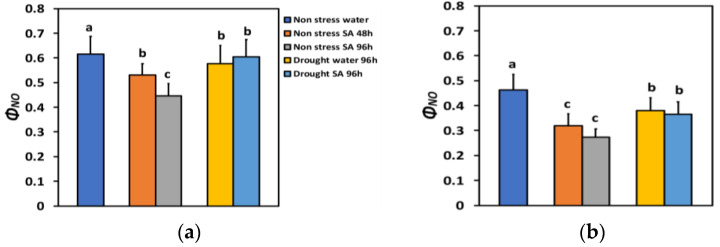
The quantum yield of non-regulated energy loss in PSII (Φ*_NO_*) at 200 μmol photons m^−2^ s^−1^ (LL) (**a**) or 900 μmol photons m^−2^ s^−1^ (HL) (**b**) of basil plants, under non-stressed control conditions (water sprayed), and 48 h and 96 h after the spray with 1 mM SA, or 96 h after withholding the irrigation on the water-sprayed or SA-sprayed plants. Error bars are standard deviations (SD). Different lowercase letters express significant difference.

The quantum yield of non-regulated energy loss in PSII (Φ*_NO_*) under NS conditions and LL decreased after 48 h of SA spraying by 14% and after 96 h of SA spraying by 28% (Figure 4a), while under HL, it decreased more (−31% after 48 h and −41% after 96 h of SA spraying) (Figure 4b). However, under HL, there was not any significant difference between the two time periods (Figure 4b).

Φ*_NO_* under LL and MiDS in the water-sprayed plants decreased by 6% and by 2% in the SA-sprayed ones, compared to NS water-sprayed plants (Figure 4a). Under HL in MiDS basil plants, a higher decrease in Φ*_NO_* occurred in both water-sprayed plants (−18%) and SA-sprayed ones (−21%), compared to NS water-sprayed plants (Figure 4b). However, in MiDS plants under both LL (Figure 4a) and HL (Figure 4b), there was not any significant difference between the two different treatments.

### 2.3. Electron Transport Rate and Photoprotective Heat Dissipation under Non-Stress or Mild Drought Stress with or without Salicylic Acid

**Figure 5:**
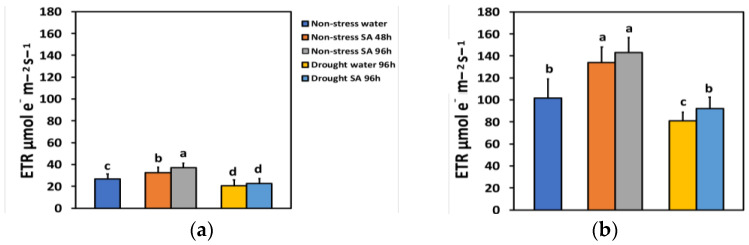
The electron transport rate (ETR) at 200 μmol photons m^−2^ s^−1^ (LL) (**a**) or 900 μmol photons m^−2^ s^−1^ (HL) (**b**) of basil plants, under non-stressed control conditions (water sprayed), and 48 h and 96 h after the spray with 1 mM SA, or 96 h after withholding the irrigation on the water-sprayed or SA-sprayed plants. Error bars are standard deviations (SD). Different lowercase letters express significant difference.

The electron transport rate (ETR) under NS conditions and LL 48 h after spraying with SA increased by 21%, while after 96 h, ETR showed a time-dependent increase by 39% (Figure 5a). Under high light (HL) and NS conditions, ETR increased even more after SA spraying (31% after 48 h and 40% after 96 h) (Figure 5b). However, there was not any significant difference between the two treatments (Figure 5b).

ETR under MiDS conditions and LL decreased significantly compared to NS conditions in both water-sprayed (−23%) or SA-sprayed (−16%) plants but without any significant difference between the two treatments (Figure 5a). In contrast, under HL, despite a significant decrease in ETR under MiDS in water-sprayed (−21%) plants, the basil plants that were sprayed with 1 mM SA retained the same ETR with NS (control) plants (Figure 5b). Salicylic acid (SA)-sprayed basil plants retained significantly higher ETR (+14%) compared to water sprayed, under MiDS treatment (Figure 5b).

**Figure 6:**
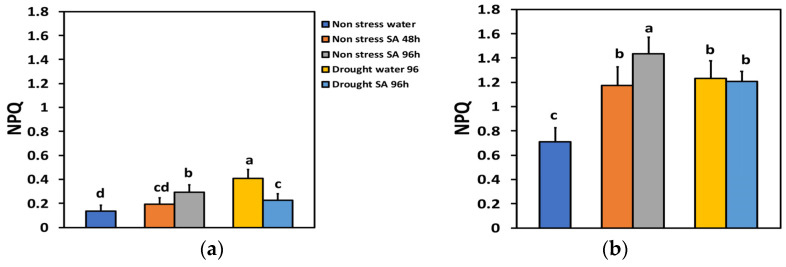
The non-photochemical quenching (NPQ) at 200 μmol photons m^−2^ s^−1^ (LL) (**a**) or 900 μmol photons m^−2^ s^−1^ (HL) (**b**) of basil plants, under non-stressed control conditions (water sprayed), and 48 h and 96 h after the spray with 1 mM SA, or 96 h after withholding the irrigation on the water-sprayed or SA-sprayed plants. Error bars are standard deviations (SD). Different lowercase letters express significant difference.

The parameter of non-photochemical quenching (NPQ), characterizing the photoprotective heat dissipation under NS conditions and LL, did not change significantly 48 h after SA spraying, but after 96 h, it increased by 114% (Figure 6a). Under HL and NS conditions, 48 h after spraying with SA, NPQ increased by 65%, and after 96 h, by 102% (Figure 6b). Under MiDS conditions and LL in water-sprayed leaves, NPQ increased by 199% while in SA-sprayed ones, by 67%, displaying a 44% less NPQ value from the water-sprayed MiDS leaves (Figure 6a). In contrast, under MiDS and HL, NPQ increased almost the same in both water-sprayed (74%) and SA-sprayed (70%) plants, displaying no significant difference between them (Figure 6b).

### 2.4. Open Photosystem II Reaction Centers and Their Efficiency under Non-Stress or Mild Drought Stress with or without Salicylic Acid

**Figure 7:**
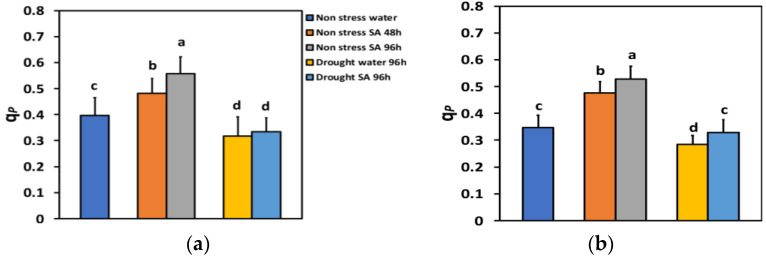
The fraction of open reaction centers of PSII (q*p*) at 200 μmol photons m^−2^ s^−1^ (LL) (**a**) or 900 μmol photons m^−2^ s^−1^ (HL) (**b**) of basil plants, under non-stressed control conditions (water sprayed), and 48 h and 96 h after the spray with 1 mM SA, or 96 h after withholding the irrigation on the water-sprayed or SA-sprayed plants. Error bars are standard deviations (SD). Different lowercase letters express significant difference.

The percentage of open reaction centers (q*p*) under NS conditions and LL 48 h after SA spray increased by 21% and 96 h after SA spray, by 41% (Figure 7a), showing a time-dependent increase. Under NS conditions and HL, q*p* increased even more after SA spray (+37% after 48 h and +52% after 96 h) (Figure 7b).

Under MiDS conditions and LL, q*p* in water-sprayed leaves decreased by −20%, while in SA-sprayed ones, by −16%, but without any significant difference between the two treatments (Figure 7a). In contrast, under MiDS and HL, q*p* decreased by −18% in water-sprayed leaves, while in SA-sprayed ones, by −5%, displaying a significant (+16%) higher percentage of open reaction centers than the water-sprayed leaves (Figure 7b).

**Figure 8:**
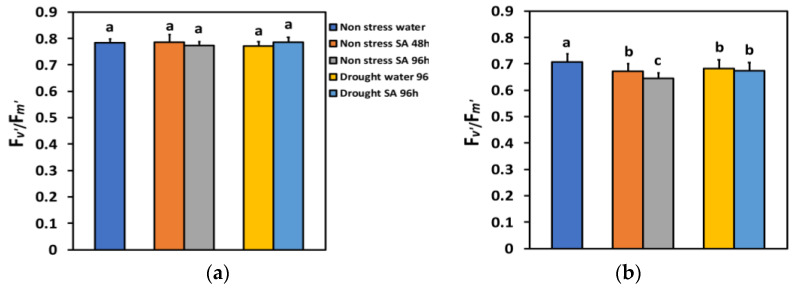
The efficiency of the open reaction centers of PSII (F*v*’/F*m*’) at 200 μmol photons m^−2^ s^−1^ (LL) (**a**) or 900 μmol photons m^−2^ s^−1^ (HL) (**b**) of basil plants, under non-stressed control conditions (water sprayed), and 48 h and 96 h after the spray with 1 mM SA, or 96 h after withholding the irrigation on the water-sprayed or SA-sprayed plants. Error bars are standard deviations (SD). Different lowercase letters express significant difference.

The efficiency of the open PSII reaction centers (F*v*’/F*m*’) in LL under NS or MiDS conditions remained unchanged (Figure 8a). F*v*’/F*m*’ in HL under NS conditions 48 h after the spray with SA decreased by −5% and 96 h after SA spray by −9%, showing a time-dependent decrease (Figure 8b). F*v*’/F*m*’ under MiDS conditions and HL in water-sprayed leaves decreased by −3%, while in SA-sprayed ones, by −5%, but without any significant difference between the two treatments (Figure 8b).

### 2.5. Photosystem II Excitation Pressure and Excess Excitation Energy under Non-Stress or Mild Drought Stress with or without Salicylic Acid

**Figure 9:**
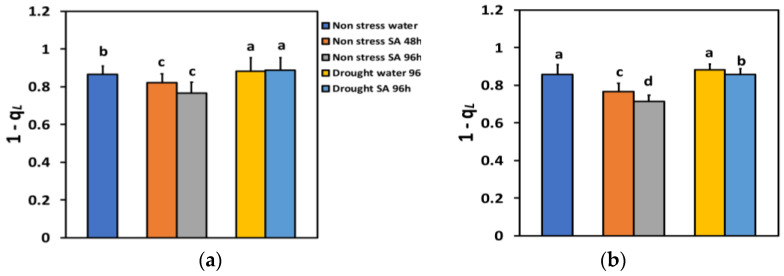
The excitation pressure at PSII (1-*qL*) at 200 μmol photons m^−2^ s^−1^ (LL) (**a**) or 900 μmol photons m^−2^ s^−1^ (HL) (**b**) of basil plants, under non-stressed control conditions (water sprayed), and 48 h and 96 h after the spray with 1 mM SA, or 96 h after withholding the irrigation on the water-sprayed or SA-sprayed plants. Error bars are standard deviations (SD). Different lowercase letters express significant difference.

The excitation pressure at PSII (1-*qL*), based on the “lake” model for the photosynthetic unit, under NS conditions and LL, decreased by −5% 48 h after the spray with SA, and by −12% 96 h after SA spray (Figure 9a), but without any significant time-dependent difference. Under NS conditions and HL, the excitation pressure at PSII (1-*qL*), 48 h after the spray with SA decreased by −11%, and 96 h after SA spray by −17%, showing a time-dependent decrease (Figure 9b).

Under MiDS conditions and LL, excitation pressure at PSII (1-*qL*), in both water-sprayed and SA-sprayed leaves, increased by +2%, without any significant difference between the two treatments (Figure 9a). In contrast, under MiDS and HL, 1 *qL* in water-sprayed leaves remained unchanged compared to control plants, but in SA-sprayed leaves, decreased by −2% compared to control plants (Figure 9b).

**Figure 10:**
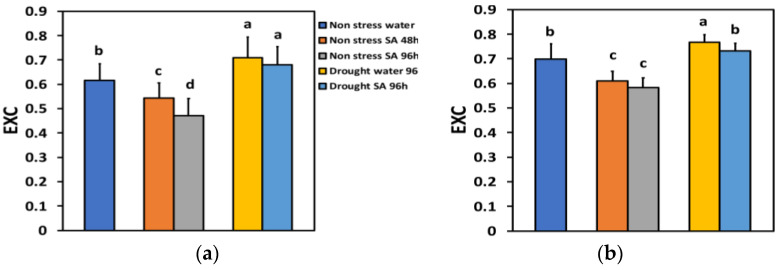
The excess excitation energy at PSII (EXC) at 200 μmol photons m^−2^ s^−1^ (LL) (**a**) or 900 μmol photons m^−2^ s^−1^ (HL) (**b**) of basil plants, under non-stressed control conditions (water sprayed), and 48 h and 96 h after the spray with 1 mM SA, or 96 h after withholding the irrigation on the water-sprayed or SA-sprayed plants. Error bars are standard deviations (SD). Different lowercase letters express significant difference.

The excess excitation energy at PSII (EXC) under NS conditions and LL 48 h after SA spraying decreased by −12% and after 96 h, by −23%, showing a time-dependent decrease (Figure 10a). Under NS conditions and HL, EXC decreased by −13% 48 h after SA spraying and after 96 h, by −17%, but without any significant difference between the two treatments (Figure 10b).

Under MiDS conditions and LL, EXC increased in both water-sprayed (+15%) and SA-sprayed (+10%) leaves, but without any significant difference between the two treatments (Figure 10a). In contrast, under MiDS and HL, EXC in water-sprayed leaves increased by +26%, but in SA-sprayed leaves, remained unchanged compared to control plants (Figure 10b).

### 2.6. Correlation of Non-Photochemical Quenching with the Quantum Yield of Non-Regulated Energy Loss in PSII

**Figure 11:**
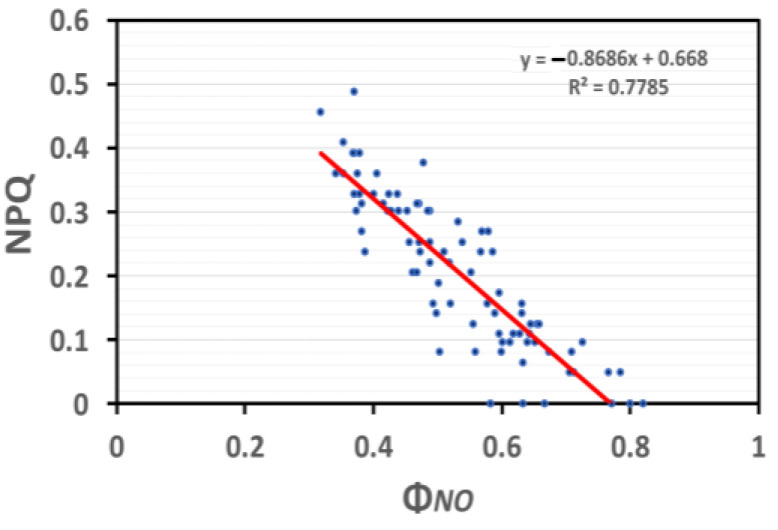
Correlation analysis between NPQ and Φ*_NO_* of basil plants under NS conditions and LL (based on data of Figure 4a and Figure 6a, respectively). Each blue dot represents the paired measurement of the variables, while the red line is the regression line that shows the relationship between the two variables.

In SA-sprayed basil plants under NS conditions, the induction of NPQ was significantly correlated (*p* < 0.001, R^2^ = 0.78) (Figure 11) to the quantum yield of non-regulated energy loss in PSII (Φ*_NO_*), which is relevant to the quantity of singlet oxygen (^1^O_2_) generation.

### 2.7. Heterogeneity of Photosystem II Function under Non-Stress or Mild Drought Stress with or without Salicylic Acid

**Figure 12:**
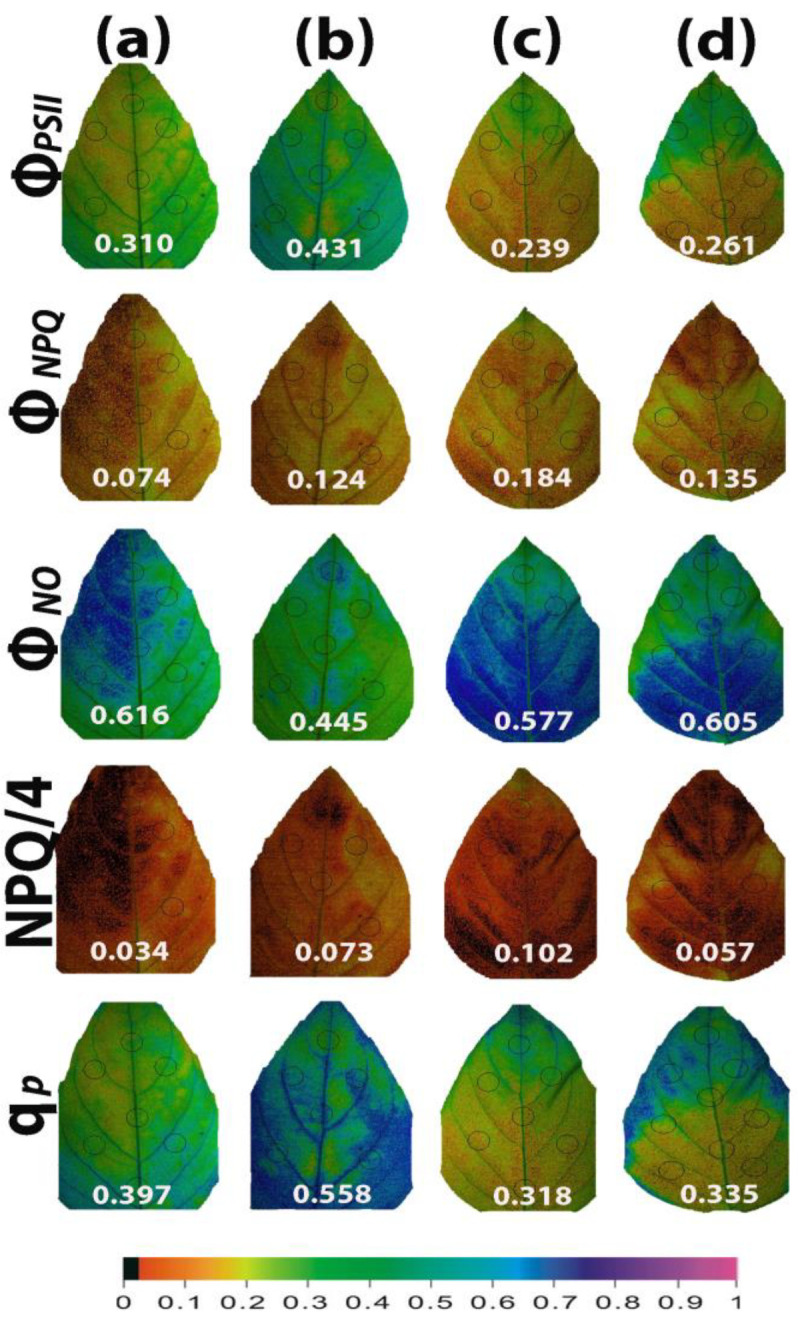
Illustrative color-coded whole leaf images of the functional PSII heterogeneity of the parameters Φ*_PSII_*, Φ*_NPQ_*, Φ*_NO_*, NPQ/4, and q*p*, evaluated at 200 μmol photons m^−2^ s^−1^, under non-stressed control conditions (water sprayed) (**a**), 96 h after the spray with 1 mM SA (**b**), after withholding the irrigation for 96 h on the water-sprayed plants (**c**), or after withholding the irrigation for 96 h on the SA-sprayed plants (**d**). A color code at the bottom, indicating the corresponding parameter values along the whole leaf area as well as the average whole leaf value, is provided.

Color-coded pictures of the whole leaf area, obtained at 200 μmol photons m^−2^ s^−1^ by the method of chlorophyll fluorescence imaging, for the parameters of Φ*_PSII_*, Φ*_NPQ_*_,_ Φ*_NO_*, NPQ/4, and q*p*, revealed a leaf PSII functional heterogeneity (Figure 12). This PSII functional heterogeneity was evident even in water-sprayed (control) leaves, with the leaf areas showing decreased Φ*_PSII_* values, which could not be compensated by the corresponding increases in Φ*_NPQ_*, resulting in increased Φ*_NO_* at the same leaf areas (Figure 12a). The leaf area with the low Φ*_PSII_* values was matched with the area with the low q*p* values (Figure 12a). The leaf PSII functional heterogeneity was less evident 96 h after the spray with 1 mM SA (Figure 12b), implying that SA enhanced PSII functionality mainly in the leaf areas with decreased performance in the light energy use. However, the high increase in the whole leaf q*p* value (+41%) was also due to improved q*p* values of the whole leaf area (Figure 12b). After withholding the irrigation for 96 h, leaf PSII functional heterogeneity of the water-sprayed plants increased (Figure 12c). In SA-sprayed leaves, PSII functionality improved from the leaf tip to the leaf base (Figure 12d).

### 2.8. Chloroplast Ultrastructure under Non-Stress or Mild Drought Stress with or without Salicylic Acid

**Figure 13:**
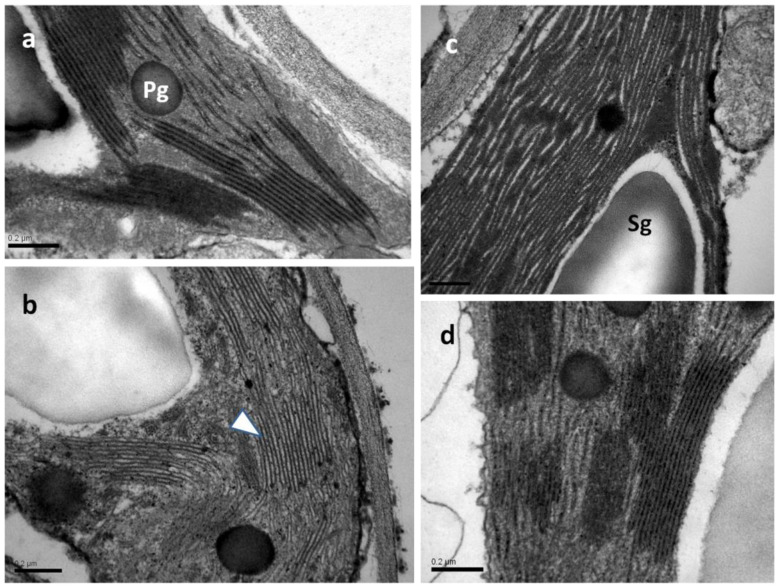
TEM micrographs of chloroplast sections depicting thylakoid organization in water-sprayed (**a**,**b**) and SA-sprayed (**c**,**d**) leaves, either NS (**a**,**c**) or MiDS (**b**,**d**). In NS water-sprayed and SA-sprayed leaves (**a**,**c**), as well as under MiDS, SA-sprayed leaves (**d**), thylakoids appear tightly packed. The impact of MiDS on water-sprayed leaves is manifested by a dilation of thylakoids (**b**), the content of which appears electron transparent (arrowhead in (**b**)). Pg: plastoglobuli, Sg: starch grain, scale bars: 0.2 μm.

In all samples observed by transmission electron microscopy (TEM), the chloroplasts exhibited integral plastid envelopes, well-developed frets and grana thylakoids, plastoglobuli, and starch grains (Figure 13). In both water-sprayed and SA-sprayed NS leaves (Figure 13a,c), as well as in SA-sprayed MiDS leaves (Figure 13d), thylakoids were tightly packed with a prominent electron-dense content. On the contrary, in chloroplasts of water-sprayed MiDS leaves, the thylakoids appeared slightly dilated, including electron-transparent content (Figure 13b).

## 3. Discussion

With the progressively increasing water-deficit stress episodes due to climate change [1,3,81], research on crop tolerance to water deficit is fundamental to alleviate expected pressures on water resources [82]. Foliar application of SA has been shown to alleviate the adverse effects of drought stress and salinity by improving osmotic potential, reducing membrane damage, regulating stomatal conductance and transpiration rate, and enhancing photosynthesis [54,55]. Its significance lies particularly in its ability to modulate responses to both biotic and abiotic stresses, functioning as a plant growth regulator signalling molecule and an antioxidant [51,52,54,55,83]. Notably, SA accumulation during drought stress in *Avena sativa* affects photorespiration, stomatal opening, and antioxidant defenses before any significant change in leaf water content occurs [53]. Similarly, in our experiment under MiDS, the leaf water content of SA-sprayed leaves did not differ compared to NS in either water-sprayed or SA-sprayed plants (Figure 1b). However, there were noticeable changes in PSII functioning.

PSII efficiency in SA-sprayed leaves under NS conditions, evaluated at both LL (Figure 2a) and HL (Figure 2b), increased significantly with a parallel significant decrease in the excitation pressure at PSII (1-*qL*) (Figure 9a,b) and the excess excitation energy (Figure 10a,b). A decreased 1-*qL*, as observed in SA-sprayed leaves at both LL and HL, under NS conditions (Figure 9a,b), equals to a more oxidized quinone A (Q_A_) (Figure 7a,b), corresponding to decreased stomatal opening [79,84,85]. The oxidation state of Q_A_ is considered to reflect the equilibrium between excitation energy at PSII and the rate of the Calvin–Benson–Bassham cycle [85]. Plants with increased levels of NPQ display less stomatal opening in response to light, developing a 25% decline in water loss per CO_2_ fixed [86]. Stomatal opening is rather coordinated by the redox state of the plastoquinone (PQ) pool instead of the Calvin–Benson–Bassham cycle or the amount of CO_2_ fixation [87].

Increased values of Φ*_PSII_* can be ascribed either to an increased percentage of open PSII reaction centers (q*p*), and/or to an improved efficiency of the open PSII reaction centers (*Fv*’/Fm’) [88]. The enhancement of Φ*_PSII_* in SA-sprayed leaves under NS conditions evaluated at both LL (Figure 2a) and HL (Figure 2b) was due to a higher percentage of open PSII reaction centers (q*p*) (Figure 7a,b), since there was a decreased efficiency of the reaction centres at HL (Figure 8b) or no alteration of their efficiency at LL (Figure 8a).

Efforts to improve photosynthetic efficiency in crop plants are projected to enhance light energy utilization [89,90,91]. SA foliar spray under NS conditions at LL enhanced ETR by 21% after 48 h and by 39% after 96 h (Figure 5a), while under HL, ETR increased by 31% after 48 h and 40% after 96 h (Figure 5b). Particularly, an enhancement of ETR by 20% caused a yield increase in sorghum by 8% [89] and in wheat by 7.3% [91]. Even low concentrations of SA have been shown to improve the plant growth, the efficiency of the water-splitting complex, and the ETR in rice plants under Cd stress [11]. Overall, the beneficial role of SA under various abiotic stress conditions is associated with its ability to counteract oxidative damage triggered by these stressors [62,63,92], serving as an antioxidant [50,62,79] and playing a regulatory role in photosynthetic reactions [93]. The most sensitive component of the photosynthetic machinery to various abiotic stresses has been demonstrated to be PSII [21,26,27,94]. The effectiveness of chlorophyll fluorescence analysis for exploring PSII functionality under MiDS has been extensively documented [2,3,4,81,88,95].

SA is implicated in plant responses to several biotic and abiotic stresses such as pathogen infection, heat, chilling, salinity, heavy metal toxicity, and drought [62,63,64,65,66,67,68,69]. SA application improved growth and photosynthetic performance and enhanced the expression of the up-regulated genes under abiotic stress, OsDREB2A and OsSAPK8, improving drought tolerance in rice [96]. In salt-stressed cherry rootstocks, SA application increased the photosynthetic rate (Pn), the maximum efficiency of PSII photochemistry (F*v*/F*m*), and the activities of antioxidant enzymes (superoxide dismutase, peroxidase, and catalase), improving their salt tolerance [97]. Under salt stress, SA treatment protected Ethiopian mustard plants from oxidative damage by increasing antioxidant enzyme activity and modulating the cell redox balance [98], while in mungbean plants, it enhanced antioxidant enzymes and gas exchange parameters together with osmoprotectant molecules like proline [99]. Plants that enhance osmolyte accumulation e.g., proline, can compensate for cell water loss and are capable of preserving high leaf water potential, while soil water potential is low [13,100,101,102].

The SA mode of action depends significantly on various factors, including the concentration used, the duration of exposure, the environmental conditions, the plant species, and the interaction with other plant hormones like, auxin, cytokinin, jasmonic acid, ABA, and ethylene [51,103,104]. It has been shown that SA decreases ETR in tobacco [56] but enhances it in tomato plants [50]. In barley, SA decreased the efficiency of the oxygen evolving complex (OEC) in a concentration-dependent manner, resulting also in a decreased fraction of open PSII centers (q*p*) [93]. NPQ, which reveals heat dissipation of excitation energy, decreased after SA treatment in tomato plants at LL, while at HL, remained equal to control plants [50]. Activation of the NPQ mechanism to dissipate excess PSII energy in tomato plants, by the herbicide glyphosate, conferred tolerance to environmental abiotic stress [94].

In SA-sprayed basil leaves under NS conditions, the increased absorbed light energy that was allocated to photochemistry (Φ*_PSII_*) and to heat dissipation (Φ*_NPQ_*) resulted in the reduced non-regulatory loss of light energy (Φ*_NO_*). Similarly, under MiDS conditions, the increased Φ*_NPQ_* in SA-sprayed leaves overbalanced the decrease in Φ*_PSII_*, resulting in decreased Φ*_NO_*. Since Φ*_NO_* is considered to be relevant to the quantity of singlet oxygen (^1^O_2_) generation [105,106,107,108], it is suggested that in SA-sprayed leaves, the amount of ^1^O_2_ generation decreased. Preventing ^1^O_2_ generation is the crucial assignment of the photoprotective mechanism of NPQ [109,110]. The decreased generation of ^1^O_2_ is attributed to the increased NPQ that has been proposed to regulate in a way the creation of ROS [111,112,113]. Thus, it is suggested that SA induced an increase in the NPQ development, resulting in reduced ^1^O_2_ production and, simultaneously, in enhanced Φ*_PSII_* under NS conditions at both LL and HL. The induction of the NPQ mechanism that prevents the detrimental production of ROS is also regulating the photosynthetic ETR [20,111]. Nevertheless, in oregano seedlings, exogenously applied SA under NS conditions enhanced PSII functionality, only under HL and not LL conditions [79]. Under the same deficit conditions, as in the present work, SA ameliorated PSII functionality in oregano seedlings, under both LL and HL [79]. Our hypothesis that the application of SA would enhance PSII functionality in basil plants under NS conditions at both LL and HL was confirmed, but in disagreement with our hypothesis, SA ameliorated the negative impact of water deficit on PSII functionality, only at HL and not at LL conditions. Thus, the SA mode of action could depend on the plant species, its growth conditions, the light requirements of the plant species, and the environmental stress conditions.

The NPQ mechanism of photoprotection is considered to be sufficient under environmental stress conditions if it can maintain an equal percentage of open PSII reaction centers as in NS conditions [1,114,115]. Otherwise, a disproportion among the source energy and demand occurs, indicating excess excitation energy under environmental constrains [18,23,114]. The redox state of the PQ pool is known to be important for retrograde signaling [116,117,118]. Investigating the redox status of chloroplast can offer valuable information concerning plant responses to drought stress imposed on leaf photosynthesis, which is of great significance in crop plants since it significantly affects plant productivity [119]. Under MiDS conditions and HL, SA-sprayed leaves could retain the same percentage of open PSII reaction centers (q*p*) to control plants (Figure 7b) without having any significant different NPQ from water-sprayed MiDS leaves (Figure 6b). In addition, SA-sprayed leaves retained the same Φ*_PSII_* and ETR as control plants (Figure 2b). However, the NPQ photoprotective mechanism was not efficient in SA-sprayed leaves under MiDS conditions and LL (Figure 6a) since the fraction of open PSII reaction centers was significantly lower than control plants (Figure 7b). This may be due to basil plants acclimating better to HL conditions and requiring high light intensity sunlight [120,121]. NPQ is involved in the mechanism of plant acclimation to environmental constrains and has also been suggested to be a key component of the systemic acquired resistance [16,122,123,124].

## 4. Materials and Methods

### 4.1. Plant Material and Growth Conditions

Basil (*Ocimum basilicum* L.) plants, which are largely cultivated for medicinal and religious purposes [125], were obtained from the market (Garden Center Vaseiliadis) and transferred in a growth chamber (EF7; Conviron, Montreal, QC, Canada) with 22 ± 1/19 ± 1 °C day/night temperature, 55 ± 5/60 ± 5% day/night relative humidity, 200 ± 10 μmol quanta m^−2^ s^−1^ photosynthetic photon flux density (PPFD), and a 14 h photoperiod.

### 4.2. Drought Stress and Salicylic Acid Treatments

Salicylic acid (SA) was applied at 1 mM by hand spraying on non-stressed or drought-stressed basil plants, as described previously [79]. Control plants were sprayed with distilled water [79]. Each plant received 10 mL of distilled water or 10 mL of 1 mM SA. All treatments were performed with three plants and three independent biological replicates.

Basil plants were watered to full soil water capacity, and then by withholding the irrigation for 96 h, mild drought stress (MiDS) was produced, so the basil plants retained 60% soil volumetric water content (SWC) of the well-watered (control) plants. This mild drought-stress (MiDS) treatment was selected in order to achieve the same drought-stress conditions that were applied previously in oregano plants [79].

### 4.3. Soil Water Status and Leaf Water Content

Soil volumetric water content (SWC) was measured with the ProCheck device that was connected to a 5TE sensor (Decagon Devices, Pullman, WA, USA). The water content of basil leaves was estimated using the electronic moisture balance (MOC120H, Shimadzu, Tokyo, Japan).

### 4.4. Chlorophyll Content and Chlorophyll Fluorescence Analysis

Relative chlorophyll content was measured photometrically with a portable Chlorophyll Content Meter (Model Cl-01, Hansatech Instruments Ltd., Norfolk, UK) and expressed in relative units [126].

Photosystem II photochemistry was measured using the modulated Imaging-PAM Fluorometer M-Series (Heinz Walz GmbH, Effeltrich, Germany) according to our previously published protocol [127]. Basil plants under NS or MiDS conditions, sprayed with water or SA, were dark-adapted for 30 min before measurements. Measurements were conducted on basil leaves 48 and 96 h after the foliar spray by water or 1 mM SA with 200 μmol photons m^−2^ s^−1^ (LL) actinic light (AL) or 900 μmol photons m^−2^ s^−1^ (HL). The illumination time with AL was 5 min, and during actinic light application, saturating pulses (SPs) of 6000 μmol photons m ^−2^ s^−1^ were applied every 20 s. Acclimation time to the two levels of AL were seconds. Measurements were repeated with basil plants under MiDS conditions that were created by withholding the irrigation for 96 h. The chlorophyll fluorescence parameters that were estimated using the Win software (version 2.32) (Heinz Walz GmbH, Effeltrich, Germany) are described with their formulas in Supplementary Table S1. Color-coded images of selected chlorophyll fluorescence parameters from non-stressed (NS) or mild drought-stressed (MiDS) representative leaves are also presented.

### 4.5. Transmission Electron Microscope Observations

Mature leaves from water-sprayed or SA-sprayed plants under NS or MiDS conditions were cut with razor blades in pieces ~2 × 2 mm^2^, which were immediately fixed in 3% glutarhaldehyde in 50 mM sodium cacodylate buffer (pH 7) for 4 h at room temperature. After 3 × 15 min rinses in the same buffer, the samples were post-fixed overnight in 1% osmium tetroxide at 4 °C. After rinsing as above, the samples were dehydrated in an acetone series, treated 2 × 20 min with propylenoxide at 4 °C, and finally embedded in Spurr’s resin. Ultrathin sections (~70 nm) were double stained with uranyl acetate and lead citrate and examined with a JEOL JEM 1011 transmission electron microscope (TEM) at 80 kV.

### 4.6. Statistics

The statistical analysis was performed in R software, version 3.4.3 (R core team, 2023). The dataset was first checked for normality and heteroskedasticity with the Shapiro–Wilk and Levene’s test (“car” package). Data that did not meet the assumptions were transformed logarithmically to satisfy these conditions for statistical analysis. Consequently, two-way ANOVA was conducted for each photosynthetic parameter to assess the effect of SA and drought on each of them. Given the significant overall effect of the factors, post-hoc analysis was performed with the Tukey test (“multcomp” package).

## 5. Conclusions

In conclusion, this study demonstrated the influence of SA application on PSII functionality of basil plants under non-stress (NS) or mild drought-stress (MiDS) conditions. Foliar SA spraying enhanced PSII function by inducing the non-photochemical quenching (NPQ) mechanism, thus dissipating the excess excitation energy under NS conditions. Under MiDS and high light (HL), the redox status of the plastoquinone pool (q*p*) in SA-sprayed leaves initiated the acclimation response to MiDS by retaining the same electron transport rate (ETR) to control plants. These results provide novel insights into the mechanisms of SA action on PSII function and may be meaningful to the application of SA in agriculture to promote global food productivity.

## Data Availability

The data presented in this study are available in this article.

## Supplementary Materials

The following Supporting Information can be downloaded at: https://www.mdpi.com/article/10.3390/ijms25115728/s1. References [128,129,130,131] are cited in Supplementary S1 file.
