# Supplementary material for: Mechanistic Insights on Salicylic Acid-Induced Enhancement of Photosystem II Function in Basil Plants under Non-Stress or Mild Drought Stress
Source: Int J Mol Sci. 2024 May 24;25(11):5728. doi: 10.3390/ijms25115728 (PMC11171592; doi:10.3390/ijms25115728)
Supplement: Supplementary file 1 [file ijms-25-05728-s001.zip › ijms-2966184-supplementary.pdf]

Table S1. Definitions of the chlorophyll fluorescence parameters used in the experiments.

Parameter	Definition	Calculation
Φ_{PSII}	Effective quantum yield of PSII photochemistry	$(Fm' - Fs)/Fm'$ [80,88]
Φ_{NPQ}	Quantum yield of regulated non-photochemical energy loss in PSII	$Fs/Fm' - Fs/Fm$ [128]
Φ_{NO}	Quantum yield of non-regulated energy loss in PSII	Fs/Fm [128]
Fv'/Fm'	Efficiency of the open PSII reaction centers	$(Fm' - Fo')/Fm'$ [88]
ETR	Electron transport rate	$\Phi_{PSII} \times PAR \times c \times abs$, where PAR is the photosynthetically active radiation, c is 0.5, and abs is the total light absorption of the leaf taken as 0.84 [129]
q*p*	Photochemical quenching, representing the redox state of quinone A (Q_A), or in other words the fraction of open PSII reaction centers based on the "puddle" model for the photosynthetic unit	$(Fm' - Fs)/(Fm' - Fo')$ [88]
NPQ	Non-photochemical quenching reflecting the dissipation of excitation energy as heat	$(Fm - Fm')/Fm'$ [130]
EXC	Excess excitation energy	$(Fv/Fm - \Phi_{PSII})/Fv/Fm$ [131]
1-qL	The fraction of closed PSII reaction centres based on the "lake" model for the photosynthetic unit	$1- (q_p \times F_o'/F_s)$ [80]
